# Relative Preference for In-Person, Telehealth, Digital, and Pharmacologic Mental Health Care After the COVID-19 Pandemic: Cross-Sectional Questionnaire Study

**DOI:** 10.2196/54608

**Published:** 2025-02-13

**Authors:** E Marie Parsons, Zoë G Figueroa, Michele Hiserodt, Talea Cornelius, Michael W Otto

**Affiliations:** 1 Department of Psychological and Brain Sciences Boston University Boston, MA United States; 2 Center for Behavioral Cardiovascular Health Columbia University Irving Medical Center New York, NY United States

**Keywords:** stigma, digital CBT, age, generalized anxiety disorder, insomnia, adult, telehealth, digital health

## Abstract

**Background:**

Most adults and children in the United States fail to receive timely care for mental health symptoms, with even worse rates of care access for individuals who belong to racial and ethnic minority groups. Digital (ie, app-based) care has proven to be an efficacious and empirically supported treatment option with the potential to address low rates of care and reduce care disparities, yet little is known about the relative preference for such treatment. Furthermore, the rapid adoption of telehealth care during the COVID-19 pandemic may have shifted care preferences.

**Objective:**

This study aimed to examine relative treatment preferences for 4 different types of mental health care: in-person psychological care, telehealth psychological care, digital treatment, or pharmacologic care. Care preferences were also examined relative to potential predictors of care use (ie, gender, race, age, stigma, discrimination, and level of shame).

**Methods:**

In this cross-sectional online survey study of adults (N=237, mean age 35 years, range 19-68 years), we ranked 4 mental health care modalities based on care preference: (1) in-person care, (2) telehealth care, (3) digital care, and (4) pharmacologic care. Preference for treatment modality was assessed based on vignette presentation for generalized anxiety disorder and insomnia. In addition, participants completed self-report questionnaires for demographics, symptom severity, and psychological and stigma-related variables.

**Results:**

We found no difference in overall preference for in-person versus both telehealth and digital care. For both generalized anxiety disorder and insomnia, participants preferred in-person care to telehealth care, although this finding was attenuated amongst older participants for insomnia treatment. Participants’ depressed mood was associated with a greater relative preference for pharmacologic care. There was no evidence of differential preference for digital care according to demographics, symptom severity, or psychological and stigma-related variables.

**Conclusions:**

These results indicate that digital care now competes well in terms of treatment preference with in-person, telehealth, and pharmacologic treatment options.

## Introduction

The majority of adults and children in need of mental health services fail to receive care, with an intensification of this failure for individuals from racial and ethnic minority groups [1-4]. As a strategy to meet care needs, empirically supported digital treatment (treatments presented through a computer program or an app) has been recommended as a strategy to reduce stigma and cost and as an early modality in a stepped care model [5-8]. Meta-analyses of outcomes for digital care, and digital cognitive behavior therapy (dCBT), in particular, support this strategy as both an efficacious and cost-effective approach for addressing care needs [9-11]. Yet, little research has been done to evaluate whether dCBT addresses the attitudinal and structural barriers that prevent the initiation of mental health care. For example, data from respondents with *DSM-IV* disorders identified as part of the National Comorbidity Survey Replication revealed that the desire to manage symptoms on one’s own, a facet of self-stigma, was the most common (72.6%) reason for not seeking treatment, and was followed by structural barriers such as cost, transportation, and care availability identified by 22.2% of respondents [12]. As such, there is a potential for fully autonomous dCBT to provide services for these individuals who are reluctant to seek in-person care [13] and those who face barriers to in-person care. In addition, because individuals from ethnic and racial minority groups have been found to perceive greater barriers and stigma for mental health treatment [2,14-16], there is a potential for digital treatments to help address these disparities in care by providing an alternative to specialty care clinics [17].

There is support for the hypothesis that digital treatment can indeed address some of the perceived barriers to treatment [18]. A previous study examined preference for digital or in-person treatment using 2 samples: undergraduate students and patients recruited from primary health care clinics. In both samples, there was a greater overall preference for in-person than digital treatment, but there was a significantly greater preference for digital treatment for those with higher levels of help-seeking self-stigma [18]. Greater preferences for digital treatment options have also been found for select groups, such as first-year college students [19]. Indeed, younger age and female sex, as well as treatment history and symptom levels, have been linked to a preference for digital treatment versus in-person treatment [20-22]. Yet, the care landscape appears to be changing rapidly. The COVID-19 pandemic hastened the acceptance of remote care options by both providers and consumers, with the rapid adoption of telehealth care across medical clinics [23,24], including mental health specialty clinics [25,26].

The purpose of this study was to examine relative treatment preferences for 4 different types of mental health care: in-person psychological care, telehealth psychological care (ie, provider and patient meeting online through a video conferencing platform), digital care (specifically, app-based treatment), or pharmacologic care. Care preferences were also examined relative to potential predictors of care use: gender, race, age, (self and public) stigma, discrimination, and level of shame. We conducted a large-scale online survey (through Amazon’s Mechanical Turk [MTurk]) to assess preference for care based on the presentation of patient vignettes for generalized anxiety disorder (GAD) and insomnia, 2 common disorders with documented associations between self-stigma and care-seeking, for which each modality of care studied has documented efficacy [2,27-31]. We also examined whether treatment history [21] or degree of affective and insomnia symptoms influenced the care preference ratings for the vignettes.

## Methods

### Participants

**Figure 1:**
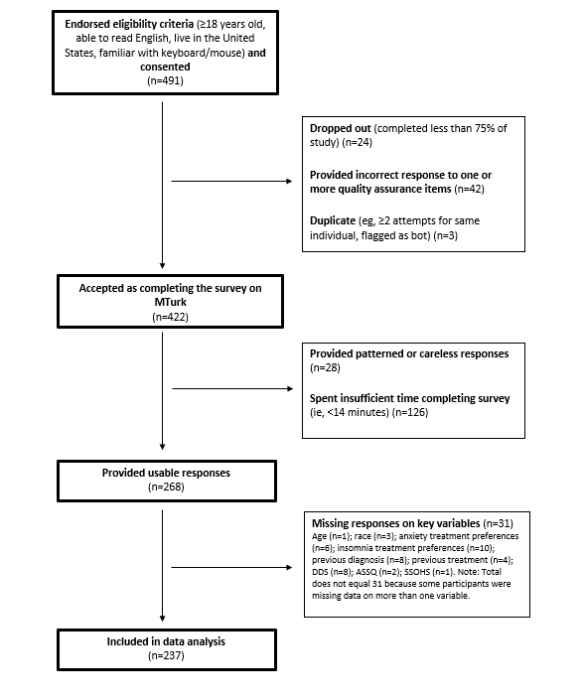
The flow of data from consent to analysis. ASSQ: Acculturative Stress Sensitivity Scale; DDS: Devaluation-Discrimination Scale; SSOHS; Self-Stigma of Seeking Help Scale.

In order to be eligible for enrollment, participants had to be 18 years of age or older and had to be able to understand English. To be included in the data analysis, participants also had to meet data quality standards appropriate for fully remote studies [32,33]. A total of 491 unique participants consented to the study, yet only 268 provided usable data, and 237 provided complete data on all variables and were included in the analysis (Figure 1 shows a diagram depicting the flow of data from consent to data analysis).

Participant ages ranged from 19 to 68 years, with a mean age of 35.41 (SD 10.53) years. In terms of the range of scores, the lowest quartile of ages reported ranged from 21 to 28 years, and the highest quartile ranged from 39 to 68 years. The majority of participants identified as male (141/237, 59.5%), 40.5% (96/237) as female, and none identified with other gender options. For race and ethnicity, 65.4% (155/237) of respondents identified as White, 20.7% (49/237) identified as Black or African American, 5.9% (14/237) identified as Asian or Asian American, 2.5% (6/237) identified as Latino/a/x or Spanish origin, 1.7% (4/237) identified as American Indian or Alaskan Native, 2.5% (6/237) selected other race or ethnicity, 0.8% (2/237) selected biracial or multiracial, 0.4% (1//237) selected Arab American/Middle Eastern or North African.

### Procedure

The survey was administered on Qualtrics between July 19, 2021, and July 26, 2021. Participants were recruited through Amazon’s MTurk platform and received US $3 for study participation. Participants were asked to provide demographic information. Then, participants read 2 vignettes and ranked mental health treatment preference options pertaining to each diagnosis presented in the vignette, with the following question: “Imagine that you have symptoms of [anxiety/insomnia] like [name of the person in the vignette] and want to seek therapy for these symptoms, please rank order the following imagining that you will be interested in seeking treatment for anxiety/insomnia at a later point.” The use of vignette-based questions is common in preference research [34] and has been shown to be effective in providing an environment to share real-life experiences participants may otherwise be reluctant to offer [35]. In this study, 2 vignettes, one depicting GAD and the other, insomnia, were presented in a counterbalanced fashion to evaluate preference for in-person care, telehealth care, digital care, or pharmacologic care (more details in Multimedia Appendix 1). After completing vignette ratings, participants then completed questionnaires on their present levels of generalized anxiety and insomnia (GAD and Sleep Condition Indicator-8 [SCI-8]) and current interest in treatment for these concerns, presented in counterbalanced order. Participants then completed questionnaires about additional experiences that may impact treatment preferences (Patient Health Questionnaire-8 [PHQ-8], Structural Barriers, Shame Inventory [SI], Devaluation-Discrimination Scale [DDS], Self-Stigma of Seeking Help Scale [SSOSH], Brief Perceived Ethnic Discrimination Questionnaire-Community Version [Brief PEDQ-CV], and Acculturative Stress Sensitivity Scale [ASSQ]). Participants were subsequently asked to provide information on previous mental health diagnoses and whether they received treatment (8 participants indicated “declined to respond” regarding their previous mental health were coded as having no previous mental health treatment).

### Measures

#### Anxiety Symptoms and Related Treatment Preferences

The GAD-7 is a self-report measure used to assess the severity of symptoms related to GAD from the past week [36]. It was shown to have good reliability and criterion and construct validity [36]. Participants indicate agreement with 7 statements using a 4-point Likert scale from 0 (Not at all) to 3 (Nearly every day), with responses on all items summed to create a total score ranging from 0 to 21. Higher scores indicate greater anxiety. The GAD-7 had excellent internal consistency in the current sample (α=.91). An additional item was added to assess participants’ interest in treatment (yes or no), given their current level of anxiety.

#### Insomnia Symptoms and Related Treatment Preferences

The Sleep Condition Indicator-8 (SCI-8) is an 8-item scale used to screen for insomnia [37]. The scale can also be used to assist in diagnosis and for clinical and self-monitoring of changes in symptoms related to treatment [38]. It is considered reliable and valid. Participants respond using several 5-point Likert scales, all ranging from 0 to 4; all items are summed to create a total score ranging from 0 to 32. Higher scores indicate more mild symptoms, whereas scores of 16 or less reflect the recognized threshold criteria for insomnia disorder [37]. SCI-8 had good internal consistency in the current sample (α=.87). An additional item was added to assess participants’ interest in treatment (yes or no), given their current level of sleep difficulties.

#### Depression Symptoms

PHQ-8 is a self-report scale providing a reliable and valid measure of the severity of depression [39,40]. Participants are asked to respond to 8 items using a 4-point scale from 0 (Not at all) to 3 (Nearly every day); all items are summed to create a composite score ranging from 0 to 24 [39]. Higher scores indicate stronger severity. In this study, this measure had excellent internal consistency in the current sample (α=.91).

#### Structural Barriers

A 6-item Structural Barriers scale that was developed for this study was used to assess the extent different structural barriers inhibit individuals from engaging in in-person mental health treatment (ie, lack of insurance coverage, cost, unsure about where to go or whom to see, the time required or inconvenience, being unable to get an appointment, problems with childcare, transportation, or scheduling). Items for this scale were adapted from a list of barriers to treatment identified in previous research [41]. Participants respond using a 4-point Likert scale ranging from 0 (Not at all) to 3 (To a great extent). Higher scores indicate greater hesitancy to engage due to structural barriers. This measure had acceptable internal consistency in the current sample (α=.79).

#### Shame

The SI is a 3-item self-report scale used to assess how individuals experience shame in relation to global and specific life events [42]. The Shame Inventory has been found to have high internal consistency, reliability, and construct validity [42]. There are 2 parts to this scale; part 1 asks about overall shame and was the only part used in this study. Participants responded on a 5-point Likert scale from 0 to 4. Items are summed, with higher scores indicating higher degrees of shame. The internal consistency of this measure in the current sample was acceptable (α=.77).

#### Mental Health Stigma

The DDS is a 12-item self-report measure used to assess the extent to which an individual believes others will discriminate or devalue someone with a mental illness [43]. It is one of the most frequently used measures to determine the perception of social stigma pertaining to mental illness and has high internal consistency and reliability [44]. Participants respond on a 6-point Likert scale from 1 (Strongly agree) to 6 (Strongly disagree), with higher scores indicating increased perception of public stigma. This measure has excellent internal consistency in the current sample (α=.9).

The SSOSH is a 10-item scale used to measure the self-stigma associated with seeking psychological help [45]. SSOSH has strong internal consistency reliability and test-retest reliability while also supporting its validity [45]. Participants are asked to evaluate the degree to which statements describe how they might react in situations related to seeking professional health (eg, My self-esteem would increase if I talked to a therapist, I would feel inadequate if I went to a therapist for psychological help) using a 5-point Likert scale from 1 (Strongly disagree) to 5 (Strongly agree). The internal consistency of this measure, the only measure with reverse-coded items in this study, was low (α=.53).

#### Racial and Ethnic Discrimination

The Brief PEDQ-CV is a 17-item measure used to assess perceptions of racial and ethnic discrimination [46]. Participants are asked to indicate how often various situations have happened to them, based specifically on their ethnicity, using a 5-point Likert scale from 1 (Never happened) to 5 (Happened very often). Higher scores indicate a higher perception of racial and ethnic discrimination. The internal consistency of this measure in this study was excellent (α=.97).

The ASSQ is a 28-item scale used to measure an individual’s sensitivity to discrimination and acculturative stress [47]. Participants respond on a 5-point Likert scale ranging from 1 (None) to 5 (Much or very much), with higher scores indicating greater acculturative stress. The internal consistency of this measure in the current sample was excellent (α=.98).

### Data Analysis Strategy

Exploded logistic regression analyses with exact ties were estimated in SAS (version 9.4; SAS Institute) to examine systematic differences in treatment preferences (telehealth, digital, pharmacologic, and in-person therapy), with in-person therapy as the reference value. Data were structured such that each participant had 4 rows, 1 for each treatment option, and the outcome for each row was the assigned rank for that treatment option (1, 2, 3, or 4, with 1 being the most preferred and 4 being the least preferred). This was accomplished using PROC PHREG with exact ties and specifying participant ID numbers as strata to account for multiple observations.

After examining the main effects, interaction analyses were conducted to test whether participant characteristics were associated with rank choices by entering the multiplicative interaction term for each covariate separately with the nonreference choice values (ie, telehealth, app, and pharmacologic). Interactions were tested for significance using omnibus tests, such that a significant interaction indicated that this characteristic was associated with ranked choice broadly rather than examining only pairwise comparisons. However, individual *P* values for pairwise comparisons in interaction analyses are reported for completeness.

### Ethical Considerations

All procedures were approved by the university Boston University Institutional Review Board (protocol #6064X). Electronic informed consent was obtained from all participants, who were informed of their right to withdraw from the study at any time. Participants received $3 for study completion. The data presented in this article have been deidentified.

## Results

### Symptom Severity and Treatment-Seeking

In terms of disorder status and treatment history, 34.2% (81/237) of participants self-reported 1 previous mental health diagnosis, 31.7% (75/237) of participants self-reported more than 1 previous mental health diagnosis, and 34.2% (81/237) self-reported no history of mental health diagnosis. Out of the full sample, 43.5% (103/237) reported previous mental health treatment, and 3% (7/237) of participants preferred not to answer. The mean score on the GAD-7 was 9.41 (SD 5.55), and 55.3% (131/237) of participants had scores in the clinical range (scores ≥10). Correspondingly, 61.2% (145/237) of the sample indicated a current interest in receiving treatment for GAD. The mean score on the SCI was 18.69 (SD 6.65), with 59.5% (141/237) scoring in the range for likely insomnia (scores ≤16). Finally, 57.4% (136/237) of the sample indicated a present interest in receiving treatment for their insomnia symptoms.

### Treatment Preferences for Generalized Anxiety Disorder

Table 1 shows the mean treatment preference rankings for anxiety, as calculated using exploded logistic regression. There was no evidence that participants systematically preferred 1 type of treatment or tended to rank treatment options in a specific order (*χ^2^*_3_=4.25; *P*=.24). However, testing pairwise comparisons indicated that telehealth care was ranked as less preferable than in-person care (more details in Table 1).

There was no evidence that participant age (*P*=.41), gender (*P*=.51), or race or ethnicity (*P*=.74) were associated with anxiety treatment preference*.* Neither were previous mental health treatment (*P*=.61), previous mental health diagnosis (*P*=.4), baseline anxiety symptoms (*P*=.32), nor baseline sleep difficulties (*P*=.59) associated with overall treatment preferences for anxiety.

While baseline depression was not associated with treatment preference overall (*χ^2^*_3_=6.27, *P*=.10), pairwise comparisons found a significant increase in the odds of preferring pharmacologic versus in-person therapy for anxiety as depression symptoms increased (odds ratio [OR] 1.03, 95% CI 1.00-1.05; *P*=.02). Similarly, while the presence of structural barriers was not associated with treatment preference for anxiety overall, (*χ^2^*_3_=3.5; *P*=.32), pairwise comparisons found a marginally significant increase in the odds of preferring telehealth versus in-person therapy as structural barriers increased (OR 1.04, 95% CI 0.997-1.08; *P*=.07). None of the other psychological predictors were associated with treatment preference (shame: *P*=.81; devaluation discrimination: *P*=.53; self-stigma of seeking help: *P*=.54; acculturative stress sensitivity: *P*=.58; or racial and ethnic discrimination: *P*=.27).

**Table 1:** Preference ratings for each treatment modality with in-person treatment representing the comparative standard.

Ratings			*B*			OR^a^ (95% CI)			*P* value		Rank, mean (SD)			
**Ratings for the treatment of generalized anxiety disorder**														
	In-person			Ref^b^							2.30 (1.21)			
	Digital			–0.14			0.87 (0.70-1.07)		.18		2.56 (1.07)			
	Telehealth			–0.22			0.80 (0.65-0.99)		.04		2.56 (1.17)			
	Pharmacologic			–0.10			0.90 (0.73-1.11)		.33		2.59 (0.99)			
**Ratings for the treatment of insomnia**														
	In-person	Ref								2.32 (1.30)				
	Digital	0.08			1.09 (0.88-1.34)			.45		2.46 (1.03)				
	Telehealth	0.003			1.00 (0.81-1.24)			.04		2.44 (1.16)				
	Pharmacologic	–0.13			0.88 (0.70-1.08)			.22		2.78 (0.90)				

^a^OR: odds ratio.

^b^Reference.

### Treatment Preferences for Insomnia

Table 1 shows the mean treatment preference ratings for insomnia. There was no evidence that participants systematically preferred one type of treatment or tended to rank treatment options in a specific order (*χ^2^*_3_=4.4; *P*=.22).

While participant age (*P*=.08) was not significantly associated with overall treatment preferences for insomnia, pairwise comparisons indicated a significant increase in the odds of preferring telehealth versus in-person treatment as age increased (OR 1.02, 95% CI 1.00-1.03; *P*=.02). Neither gender (*P*=.55), race or ethnicity (*P*=.73), previous mental health treatment (*P*=.47), previous mental health diagnosis (*P*=.49), baseline anxiety symptoms (*P*=.38), nor baseline sleep difficulties (*P*=.90) were associated with treatment preferences for insomnia.

While baseline depression was not associated with insomnia treatment preference in terms of overall ranking patterns (*χ^2^*_3_=5.06; *P*=.17), pairwise comparisons (ie, examining the impact of depression on only the preference of pharmacologic vs in-person therapy) found a significant increase in the odds of preferring pharmacologic versus in-person therapy as depression symptoms increased (OR 1.02, 95% CI 1.00-1.05; *P*=.05). Similarly, while shame was not associated with treatment preference overall (*χ^2^*_3_=4.69; *P*=.2), pairwise comparisons indicated that there was a marginally significant increase in the odds of preferring pharmacologic versus in-person therapy as shame increased (OR 1.05, 95% CI 0.998-1.11; *P*=.06). None of the other psychological variables were associated with treatment preference for insomnia (structural barriers: *P*=.57; devaluation discrimination: *P*=.89; self-stigma of seeking help: *P*=.09; acculturative stress sensitivity: *P*=.17; and racial and ethnic discrimination: *P*=.29).

## Discussion

The purpose of this study was to examine relative treatment preferences for 4 different types of mental health care: in-person care, telehealth care, digital care, and pharmacologic care. Respondents reported familiarity with the mental health system, with almost 66% (156/237) reporting a previous mental health diagnosis and almost 44% (103/237) reporting previous mental health treatment; well over half the sample reported interest in future treatment for GAD or insomnia. For this sample, across these 2 referent disorders, there was consistent evidence that telehealth was less preferred than in-person psychological care. No significant difference in preference was found between in-person care and either digital care or pharmacologic care. Furthermore, of the range of demographic and psychological factors examined, moderation of preferences was evident only for depression and age. For rankings of both anxiety and insomnia care, the presence of greater depression was associated with a greater relative preference for pharmacologic than in-person care. Nonetheless, when all care options were considered, the degree to which pharmacologic care was selected as a first-choice option (approximately 22% (52/237) of the sample) was in strong accord with the 3-fold preference for psychosocial treatment options over pharmacologic treatment observed in a meta-analysis of this issue [34].

We did not replicate the common finding [20-22] of a greater preference for digital care among younger adults. Indeed, we found that telehealth care was relatively more preferred to in-person care among older rather than younger participants (within the age range evaluated: 19 to 68 years), although this association was found only for insomnia treatment. It is unclear whether this apparent loss of youth-based moderation of preferences for digital care is a result of the widespread adoption of more remote care options during the COVID-19 pandemic [23,24]. There are strong indications that the introduction of telehealth during the COVID-19 pandemic quickly led to a preference to continue this modality of care for mental health issues [48]. Likewise, a recent cross-sectional study reported that 73% of participants agreed or strongly agreed that the pandemic made them more open to telehealth in general [49]. A longitudinal examination of public discourse related to telehealth from March 2020 to April 2021 similarly suggests that continued widespread adoption and use of telehealth services is likely [50]. As such, one unexpected consequence of the COVID-19 pandemic may be a more widespread acceptance of telehealth and digital treatments such that some of the traditional predictors of preferences for digital treatment (ie, younger adults) are no longer relevant.

Among psychological predictors, few appeared to have a discernible effect on treatment preferences. Greater barriers showed a nonsignificant trend toward predicting a greater preference for telehealth than in-person care for GAD. More consistent effects were evident for depression severity; for treatment selection for both generalized anxiety and insomnia, a greater preference for pharmacologic relative to in-person psychological treatment was found at greater levels of depression severity.

Empirically supported digital treatment has been recommended as a strategy to reduce stigma for care [5,7], yet we did not find a relationship between stigma and preference for digital care, although we did observe a trend level association between reported shame and a relatively greater preference for telehealth than in-person therapy for insomnia. What we documented instead was acceptance of digital care that rivaled that for in-person psychotherapy, telehealth psychotherapy, or pharmacotherapy, with approximately equal numbers of participants selecting each of these choices as a first preference option. An implication of this finding is that health systems and providers may need to be prepared to provide multiple strategies for care to enhance treatment acceptance among patients.

It is important to note a number of limitations to our study findings. First, our sample was recruited through an online survey, and accordingly, only those with access to the internet were able to partake in the study. As the purpose of this study was to examine preference for treatment options that included telehealth and digital care, the assessment of individuals willing to engage with a digital assessment may have biased the results. That is, those who are unable to attain easy access to the internet may have been less inclined to prefer a telehealth or digital treatment method compared with those who already have access. Second, our results were based on responses to vignettes depicting GAD or insomnia, so treatment preferences were derived only for these specific disorders. Different relative preferences may well be present for different disorders. Third, our findings were specific to the preference for treatment initiation and do not address how preferences may change as travel or cost burdens, side effects, or other adherence issues are experienced during treatment [12,21,51]. Fourth, our preference ratings presupposed that all modalities of treatment were equally available to participants and ignored the many settings or localities where specific empirically supported mental health treatment options may not be available. Finally, we recruited a few individuals in the oldest age ranges (eg, only 3% of the sample was 65 years and older), so our findings may not apply to the preferences of older adults in this age range.

Overall, our results are suggestive of an evening-out of care preferences, where digital care competes well with other modalities of care for mental health issues, specifically GAD and insomnia. Given the many individuals who fail to receive timely care for these and other disorders [1,52], our findings provide encouragement for further digital care development, validation, and dissemination to try to address these care needs for interested patients.

## Appendix

Multimedia Appendix 1 Vignettes for provider preferences.

## Abbreviations

ASSQ: Acculturative Stress Sensitivity Scale
Brief PEDQ-CV: Brief Perceived Ethnic Discrimination Questionnaire-Community Version
dCBT: digital cognitive behavior therapy
DDS: Devaluation-Discrimination scale
GAD: generalized anxiety disorder
MTurk: Mechanical Turk
OR: odds ratio
PHQ-8: Patient Health Questionnaire-8
SCI-8: Sleep Condition Indicator-8
SI: Shame Inventory
SSOSH: Self-Stigma of Seeking Help Scale
