# Supplementary material for: Relative Preference for In-Person, Telehealth, Digital, and Pharmacologic Mental Health Care After the COVID-19 Pandemic: Cross-Sectional Questionnaire Study
Source: J Med Internet Res. 2025 Feb 13;27:e54608. doi: 10.2196/54608 (PMC11888065; doi:10.2196/54608)
Supplement: Multimedia Appendix 1 [file jmir_v27i1e54608_app1.pdf]

<u>Vignettes for Provider Preference</u>

1. Anthony is a 32-year-old business-person who has had difficulties sleeping over the last four months. It takes him a long time (over an hour many nights) to fall asleep, and then he may awaken in the middle of the night and have difficulties getting back to sleep. He feels foggy at work, and worries almost every day whether he is getting enough rest and whether he will be able to get to sleep that night. He also worries that if his sleep does not improve, he will make major mistakes at work. Additionally, because he feels more irritable most days, he feels like his relationship with his wife is disrupted.

   <u>Imagine</u> that you have symptoms of anxiety like Anthony and want to seek therapy for these symptoms, please rank order the following imagining that you will be interested in seeking treatment for anxiety at a later point: ***[1, most interested to 4, least interested]***

   - ☐ Face-to-face, in person therapy (post-COVID-19 social distancing restrictions)
   - ☐ Telemedicine (video therapy over your computer with a live therapist)
   - ☐ App-based treatment (e.g., a program on your smartphone or computer)
   - ☐ Medication treatment

2. Kayla is a 27-year-old married engineer who has experienced anxiety and worry over the past 2 years. She has felt periods of extreme muscle tension and has been easily fatigued, although she also has had difficulty falling and staying asleep. In addition, she reports irritability and a constant "edgy" and watchful feeling that has often interfered with her ability to concentrate. These feelings have been present for most of the day, more days than not. Kayla worries much of the day about multiple topics, and she recognizes that even though her worries are often unfounded, she cannot stop herself from worrying.

   <u>Imagine</u> that you have symptoms of anxiety like Kayla and want to seek therapy for these symptoms, please rank order the following imagining that you will be interested in seeking treatment for anxiety at a later point: ***[1, most interested to 4, least interested]***

   - ☐ Face-to-face, in person therapy (post-COVID-19 social distancing restrictions)
   - ☐ Telemedicine (video therapy over your computer with a live therapist)
   - ☐ App-based treatment (e.g., a program on your smartphone or computer)
   - ☐ Medication treatment
